# Between a Rock and a Hard Place: How poverty and lack of agency affect HIV risk behaviors among married women in 25 African countries: A cross-sectional study

**DOI:** 10.7189/jogh.11.04059

**Published:** 2021-10-30

**Authors:** Roya Sherafat-Kazemzadeh, Gary Gaumer, Dhwani Hariharan, Anna Sombrio, Allyala Nandakumar

**Affiliations:** Institute for Global Health and Development, The Heller School for Social Policy and Management, Brandeis University, Waltham, Massachusetts, USA

## Abstract

**Background:**

Gender inequality and poverty exacerbate the burden of HIV/AIDS among women in Africa. AIDS awareness and educational campaigns have been inadequate in many countries and rates of HIV testing and adherence to condom use remains considerably low, especially among married women. We investigate whether higher HIV knowledge is equally effective in lowering risky behaviors among groups of women with different levels of wealth and agency.

**Methods:**

Pooled data on 113 151 adult married women from Demographic and Health Surveys (DHS) in 25 African countries was used (2010 to 2016). Agency was defined as women’s ability to refuse sex and ask her partner to use a condom, plus have a role in decision making in household spending and health-related issues. The lowest tertile of DHS wealth index defined poverty. Questions about HIV prevention and mother-to-child transmission were used to create a scale for knowledge (0-5). Use of condom, HIV testing, absence of sexually transmitted disease (STD), and having one partner were dependent variables. Regression models investigated the effect of agency and knowledge as predictors of behaviors. Separate additional models were run to measure associations of each behavior with knowledge scores on groups of women divided by agency and poverty. Analyses were adjusted for demographic factors, history of pregnancy, wife-beating attitude, and country dummies.

**Results:**

Significantly higher risk and lower level of protective factors exist for poor women who lack agency. Knowledge had positive associations with a better score in behavior, higher rate of condom use and testing for HIV both among poor and not poor women. When examining compound effects of agency and poverty, absence of agency reduces the positive effect of knowledge on lowering STD rate and overall behavior score among poor women. It also nullifies the effect of knowledge on condom use in both wealth groups.

**Conclusion:**

Knowledge of HIV does not exert its potential protective effect when women live in poverty compounded with lack of agency. Success of anti-HIV programs should be tailored to dynamics of risk and sociocultural and economic context of target populations.

Women in African countries are disproportionately more vulnerable to the human immunodeficiency virus/acquired immunodeficiency syndrome (HIV/AIDS) epidemic, and the role that social and contextual factors complicate their risks has been emphasized in the literature [1]. In the early 2000s, the term ‘triple threat’ was coined to underlie the enormous challenge facing African nations: poverty, gender inequality, and the HIV/AIDS epidemic [2]. Different aspects of culture and social structure can potentiate the risk burden for women, such as lack of access to information, health care, formal education, financial opportunities, and exposure to violence in the society and at home [3]. Sociocultural forces and economic disparities manifest in various aspects of women’s lives including through gender norms and the power imbalance in sexual behaviors in the household [4]. These contextual factors have been implicated for the underachievement of HIV educational campaigns and promotion of safe sex behaviors, which require some level of self-efficacy from women to make decisions about their health and autonomy to take control of their bodies without fear of stigma in the household and in their immediate community [5]. Financial insecurity and violence against women are other external factors that force them to subjugate themselves in transactional sex for cash, financial support, or other forms of security. Without self-efficacy and agency these women will not have a chance to negotiate safe sex or to seek HIV testing and protect themselves from contracting HIV. Multiple studies have shown association between better adherence to safe sex practices and women’s empowerment in terms of education, financial independence, control over household financial decisions, and negotiating power [6-8].

Personal agency has been defined as the ability to set one’s own goals and act upon them [9]. It also requires the availability of resources for women, including accurate knowledge to make decisions. We investigate whether poverty and lack of agency exacerbate the sexual risk among married women and whether or not better knowledge is equally effective in lowering risky behaviors among groups of women with different levels of wealth and agency.

## METHODS

### Study design

This is a cross-sectional analysis of Demographic Health Surveys across 25 African countries obtained through publicly available data.

### Data

**Figure 1:**
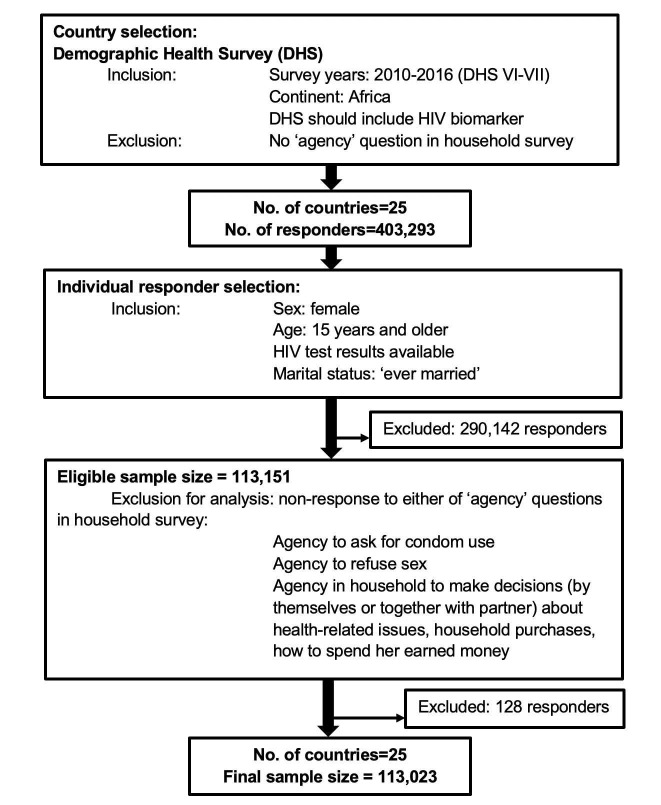
Flow-chart depicting sample selection process in this study.

We acquired the most recent data from the Demographic and Health Surveys (DHS) conducted between 2010 to 2016 (DHS VI-VII) that were accompanied by biological sample collection for HIV testing [10,11]. We merged the Female Questionnaires (individual-level) with household-level data that included demographic and socioeconomic information with HIV biomarker results of consenting participants, dropping all subjects who were missing HIV test results. We included countries whose household questionnaires included detailed questions on decision making in the household, as well as sexual behaviors. We only focused on countries from Africa and ended up with a pooled sample of 25 countries listed here: Angola, Burkina Faso, Burundi, Chad, Congo Democratic Republic, Cote d’Ivoire, Cameroon, Ethiopia, Gabon, Ghana, Gambia, Guinea, Liberia, Lesotho, Mali, Malawi, Mozambique, Niger, Namibia, Rwanda, Sierra Leone, Senegal, Togo, Zambia, and Zimbabwe. We included only married women since the questions on decision making in the household were limited to married individuals. Those responders who had missing values for any of the agency questions were removed from analyses pertaining to agency (128 individuals). A flow-chart of the sampling process is presented in **Figure 1****.**

### Agency

We conceptualized two types of agency indicators for women: 1) agency in sexual behavior by a positive answer to either of the questions on whether or not they could ask their partners to use condoms or refuse sex; 2) agency in the household related to answering if they could make decisions (by themselves or together with their partner) on any of these aspects: their health-related issues, major household purchases, and how to spend the money earned by the responder. A composite indicator of personal agency was then defined. If the woman indicated a positive response in both aspects of sexual and household agency, it was coded as “having agency”. Those women reporting agency in one or none of the three domains were coded as having ‘limited agency’.

In literature, some authors also include attitude towards wife beating as part of women’s agency and the broader concept of women’s empowerment [12,13]. We also included a composite variable for ‘rejecting wife beating’ but did not mix it with the ‘agency’ variable since this speaks towards attitude and gender norms and is not directly indicative of a ‘practice’ or power of executing one’s own decisions. The variable was assigned a value of 1 if the responder answered ‘not justified’ to all three statements: ‘wife beating is justified if she refuses to have sex’, ‘wife beating is justified if neglects the children’, and ‘wife beating is justified if she goes out without telling the husband’. A response of ‘justified’ or ‘don’t know’ was coded as zero.

### HIV-related knowledge

HIV-related knowledge was measured with three concepts: 1) comprehensive knowledge of prevention (correct answer to 2 underlying questions about use of condom and having single sexual partner as preventive measures); 2) comprehensive knowledge of mother-to-child transmission (correct answer to 3 underlying questions on possibility of transmission during pregnancy, childbirth, and nursing); and 3) correct beliefs on modes of HIV transmission. Believing that a healthy-looking person can be infected and responding correctly to at least two questions about local misconceptions (whether mosquitos, witchcraft, or sharing food could be modes of HIV transmission) counted as having a correct belief. We also created a composite knowledge score out of five underlying questions. We assigned a score of 1 for each correct answer with a final knowledge score ranging between 0-5. Responders who said they had never heard of HIV/AIDS were assigned a value of 0. A detailed description of the variables can be found in Table S1 in the **Online Supplementary Document****.**

### Risk behaviors (Outcomes of interest)

As dependent variables we chose four distinct items in the DHS questionnaire that were pertinent to behavior: 1) use of condom in last intercourse; 2) number of sexual partners including spouse over last 12 months, which was recoded as single partner if the number of partners was equal or less than 1; 3) having ever been tested for HIV; and 4) history of sexually transmitted disease (STD) over last 12 months (self-report) as an indicator for risky behavior. These variables were dichotomized and recoded so that a value of 1 indicates safe sex practices. Similar to the knowledge concept, we created a combined composite score by summing *P* values for behavior variables to generate a behavior score that ranged from 0-4. We did not impute responses for any unanswered questions. We reported the frequencies and regressions by omitting the responder from the denominator.

### Wealth

As indicators of household financial well-being, we used the DHS country-specific variable for a household wealth index (WI). We created tertiles of wealth index based on distribution of WI within each country. We identified women living in the lowest tertile of WI as ‘poor’ and those from 2^nd^ and 3^rd^ tertile were labeled as ‘not poor’.

**Analytic approach:** Ordinary least squares (OLS) and logistic regression analyses were used to examine determinants of behavior in terms of demographics and risk factors. The dependent variables for these regressions were condom use, HIV testing history, history of STD, and having a single partner, in addition to total behavior score. Models included explanatory variables for agency, knowledge, poverty, and refusal of wife beating. We controlled for demographic and socioeconomic characteristics: 1) age group (young: 15-24 years old vs old: 25 + years old), 2) education (no formal education/primary education vs secondary education and higher), 3) residential setting (urban vs rural), 4) whether or not the woman is working, 5) whether or not the woman had any pregnancy in past five years, 6) relationship to the head of household (responder herself being the head of household vs wife/co-wife vs other relationships), 7) HIV serostatus, and finally 8) dummy variables indicating the country of residence. HIV status was included because being aware of a positive status might in turn result in change in behavior or even socioeconomic status of the individual. We included pregnancy in the models since exposure to prenatal care and delivery by the help of health care professionals would change women’s access and level of knowledge and would be a source of bias in the model. Dummy variable for countries’ fixed effect would account for country-related contextual factors that are unobserved. For each of the dependent variables of interest (condom use, being tested, no history of STD, having single partner, and behavior score), the direct effect of the explanatory variables was expressed as odds ratios (for dichotomous dependent) and beta coefficient (for the behavior score). These models were run separately for the group of women who were poor (lowest tertile of WI) and the rest of sample (not poor) and the results were compared side-by-side.

In order to differentiate the effect of knowledge on behavior among different categories of agency and poverty, we classified women into four groups: 1) poor and no agency, 2) poor with some agency, 3) not poor and no agency, 4) not poor and some agency). We ran identical regression models separately for each group with the dependent variable being sexual behavior of interest, and with knowledge as independent variable. The models were controlled for covariates mentioned in the previous paragraph. We applied country-specific DHS HIV sample weights for calculation of rates and ratios, as well as regression analyses. We used Stata Statistical Software: Release 15 (Stata Corp, College Station, TX, USA) for all of the analysis.

## RESULTS

The pooled DHS data from the 25 study countries between 2010-2016 comprised 113 151 married women (25% between 15-24 years old) with an average age of 31 years and the overall HIV seropositivity rate of 5.2%. After exclusion of 128 women who had no response for the agency questions we proceeded with the analysis on a final sample of 113 023 married women.

General description of the responders: **Table 1** summarizes the general demographics of married women in this study living in households with the lowest tertile of wealth index compared to the upper two tertiles. Additionally, each group is further broken down by level of agency (no agency vs some agency). Almost one quarter of women (24.5%) across all wealth categories experience the lack of agency, but the ratio increases to one out of three among women living in poverty. Women with low agency have lower educational attainment and, if living in poverty, only 3.3% have a secondary-level education. Among all married women in this sample, 96% of those at the lowest tertile of wealth index (of their countries) live in rural areas. HIV prevalence was lower among poor women compared to their wealthier counterparts (3.9% vs 5.9%), and significantly lower in those sub-groups who lack agency.

**Table 1:** Demographic and general characteristics of the study sample

Wealth	Poor (1^st^ tertile of wealth index)		Not Poor (2^nd^ & 3^rd^ tertile of wealth index)		
**Agency level**	**Poor + No Agency**	**Poor + Some agency**	**Not poor + No agency**	**Not poor + Some sgency**	**Total**
**Number of observations**	42 385 (37.5%)		70 766 (62.5%)		113 151*,
	12 413 (11.0%)	29 930 (26.5%)	15 216 (13.5%)	55 464 (49.1%)	113 023*,
HIV prevalence (95% CI)	3.9% (3.7%, 4.1%)		5.9%§ (5.6%, 6.1%)		5.2% (5%, 5.3%)
	1.5% (1.3%, 1.8%)	4.9%§ (4.6%, 5.2%)	2.2%‡ (1.8%, 2.5%)	6.9%§ (6.6%, 7.2%)	
Average age (years) (95% CI)	30.7 (30.6, 30.8)		31.4§ (31.3, 31.5)		31.2 (31.1, 31.3)
	30.2 (30.0, 30.3)	31.0§ (30.8, 31.1)	30.2† (30.1,30.4)	31.8§ (31.7, 31.9)	
Young (15-24 years)	27.4% (26.8%, 27.9%)		23.0%§ (22.6%, 23.5%)		24.6% (24.2%, 24.9%)
	29.4% (28.4%, 30.5%)	26.5%§ (25.8%, 27.1%)	28.5% (27.5%, 29.4%)	21.5%§ (21.1%, 22.0%)	
Secondary education or higher (%)	9.5% (9.1%, 9.9%)		32.6%§ (31.9%, 33.3%)		24.4% (23.9%, 24.9%)
	3.3% (2.9%, 3.7%)	12.1%§ (11.6%, 12.7%)	11.3%§ (10.6%, 12.0%)	38.3%§ (37.6%, 39.1%)	
Working (%)	60% (59.1%, 61.0%)		59.9% (59.2%, 60.5%)		59.9% (59.4%, 60.5%)
	49.3% (47.7%, 50.9%)	64.6%§ (63.5%, 65.6%)	43.9% (42.6%, 45.2%)	64.3%§ (63.6%, 65.0%)	
Had pregnancy in recent 5 years (%)	77.2% (76.7%, 77.8%)		69.0%§ (68.5%, 69.5%)		72.0% (71.6%, 72.3%)
	77.3% (76.4%, 78.3%)	77.2% (76.6%, 77.9%)	73.4%§ (72.5%, 74.4%)	67.8%§ (67.3%, 68.4%)	
Living in rural area (%)	96% (95.6%, 96.3%)		52.2%§ (51.3%, 53.2%)		67.8% (67.1%, 68.5%)
	97.3% (96.9%, 97.8%)	95.4%§ (94.9%, 95.8%)	68.4%§ (67.0%, 69.9%)	47.9%§ (46.9%, 48.9%)	
Head of household (%)	13.7% (13.0%, 14.4%)		14.9% (14.3%, 15.5%)		14.5% (14.0%, 14.9%)
	10.8% (9.7%, 12.1%)	14.5%§ (13.8%, 15.3%)	10.2% (9.3%, 11.3%)	15.8%§ (15.2%, 16.5%)	
Wife/co-wife of head of household (%)	73.1% (72.3%, 74.0%)		71.8% (71.0%, 72.6%)		72.3% (71.7%, 73.0%)
	76.2% (74.5%, 77.8%)	72.3%§ (71.3%, 73.3%)	77.6%‡ (76.0%, 79.1%)	70.6%§ (69.7%, 71.4%)	
Other relationship to head of household (%)	13.2% (12.5%, 13.9%)		13.3% (12.8%, 13.9%)		13.3% (12.8%, 13.7%)
	13% (11.5%, 14.7%)	13.2%§ (12.4%, 13.9%)	12.1%§ (11.0%, 13.4%)	13.6%‡ (13.0%, 14.2%)	

CI – confidence interval

*The difference between total number of observations in these two rows is due to responders with missing values for agency questions who were omitted from the further analyses. Comparisons are made against the first category for each variable. Source: [14].

†*P* = 0.05.

‡*P* < 0.01.

§*P* < 0.001. Significant in unadjusted and adjusted models.

### Sexual activity and awareness of HIV/AIDS

In the pooled sample, 672 did not answer the question whether they ever had a sexual encounter and 116 stated that they have never had sex before (0.1% of responders in either poor or non-poor categories). Another preliminary question asked whether the responder had ever heard of HIV/AIDS. Overall, there was a significant contrast between the proportion of poor women with no agency who stated that they have never heard of HIV (17%) and the other three groups, especially those with agency who were not poor (2.6%). Country-specific rates are presented in Table S2 in the **Online Supplementary Document**.

### Knowledge, attitude and behaviors

**Table 2** breaks down details for HIV-related knowledge, attitudes and sexual behavior across groups of poverty and agency. The average knowledge score was lowest among poor women who lack agency. Only half of the women who lack agency knew about modes of HIV prevention or mother-to-child transmission. Women who lack agency justified wife beating at almost twice the rate of their counterparts in the same wealth level.

**Table 2:** General behavioral characteristics of women in each category of poverty and agency*

Percentage (95% CI)	Poor + No agency	Poor + Some agency	Not poor + No agency	Not poor + Some agency	Total
Number of observations	12 413	29 930	15 216	55 464	113 023
HIV prevalence (%)	1.5% (1.3%, 1.8%)	4.9%§ (04.6%, 5.2%)	2.2%§ (1.8%, 2.5%)	6.9§ (6.6%, 7.2%)	5.2% (5.0%, 5.3%)
Never heard of HIV/AIDS	17.0% (15.9%, 18.1%)	7.7%§ (7.1%, 8.3%)	10.0%§ (9.3%, 10.9%)	2.6%§ (2.4%, 2.9%)	6.4% (6.1%, 6.7%)
Comprehensive knowledge of prevention	48.0% (46.4%, 49.5%)	65.8%§ (64.9%, 66.8%)	56.2%§ (55.0%, 57.5%)	74.1%§ (73.5%, 74.7%)	67.2% (66.7%, 67.8%)
Comprehensive knowledge of mother-to-child transmission	49.8% (48.4%, 51.2%)	61.3%§ (60.5%, 62.2%)	52.3%§ (51.1%, 53.6%)	65.1%§ (64.5%, 65.8%)	61.1% (60.5%, 61.6%)
Knowledge score (0-5)	2.60 (2.55, 2.66)	3.51§ (3.48, 3.55)	3.06§ (3.01, 3.11)	3.97§ (3.95, 3.99)	3.59 (3.57, 3.61)
				
Rejection of wife beating	20.6% (19.4%, 21.8%)	40.8%§ (39.9%, 41.7%)	27.2%§ (26.1%, 28.4%)	56.4%§ (55.6%, 57.1%)	44.9% (44.3%, 45.5%)
Condom use (last encounter)	1.2% (0.9%, 1.4%)	4.6%§ (4.3%, 5.0%)	1.6%§ (1.4%, 1.9%)	7.3%§ (7.0%, 7.6%)	5.2% (5.1%, 5.4%)
Have ever been tested for HIV	23.3% (22.2%, 24.4%)	55.4%§ (54.4%, 56.4%)	34.0%§ (32.8%, 35.1%)	70.4%§ (69.7%, 71.1%)	56.7% (56.0%, 57.3%)
No STD (symptoms) over last 12 months	98.3% (98.0%, 98.6%)	98.4%† (98.2%, 98.6%)	97.9%§ (97.5%, 98.2%)	97.5%§ (97.3%, 97.7%)	97.9% (97.7%, 98.0%)
					
Having one sexual partner (or less)	99.2% (99.0%, 99.4%)	98.7%§ (98.5%, 98.8%)	99.1%§ (98.9%, 99.3%)	98.2%§ (98.1%, 98.4%)	98.6% (98.5%, 98.7%)
Behavior score (0-4)	2.17 (2.16, 2.19)	2.54§ (2.53, 2.55)	2.27§ (2.25, 2.28)	2.69‖ (2.68, 2.70)	2.54 (2.53, 2.55)

CI – confidence intervals, STD – sexually transmitted disease.

*Note: Comparisons are made against the first category for each variable. Source: [14].

†*P* < 0.05.

‡*P* < 0.01.

§*P* < 0.001. Significant in unadjusted and adjusted models.

Among these married women, the overall rate of condom use during last sexual encounter was merely 5%, with a significantly lower rate among those living in poverty and having low agency (1.2%). History of having an HIV test was on average 57% with a wide difference across groups (70% among women with agency who are not poor, vs 23% among poor women with no agency). Women with no agency, however, did better in two other aspects of behavior: they showed a lower rate of STD, and 99% reported having one (or fewer) partner(s) over the 12 months before the interview. Altogether, the overall behavior score was significantly lower in the poor group with no agency compared to any of the other three categories.

### Determinants of sexual behaviors

**Table 3** shows odds ratios for key determinants of risk behaviors in separate regression models for poor and non-poor women. The independent variables include demographic characteristics, knowledge, and whether the responder justifies wife beating. We also adjusted the models for HIV serostatus, history of pregnancy, relationship to the head of household, and dummy variables for each country.

**Table 3:** Comparison of the [direct] effects of main determinants of risk behaviors in form of odds ratio among married women who are poor (1^st^ tertile of wealth index) and not poor (2^nd^ and 3^rd^ tertiles of wealth)

	Behavior		Condom use		Been tested		No STD		Single partner	
**Odds ratio / Standard error**	**Poor**	**Not poor**	**Poor**	**Not poor**	**Poor**	**Not poor**	**Poor**	**Not poor**	**Poor**	**Not poor**
HIV seropositivity	1.119§	1.117§	3.716§	3.003§	1.350‡	1.627§	0.524‡	0.438§	0.476§	0.549§
	-0.019	-0.014	-0.342	-0.173	-0.139	-0.12	-0.111	-0.052	-0.086	-0.065
Some agency	1.061§	1.093§	1.697§	2.299§	1.436§	1.491§	1.248	0.881	0.791	0.864
	-0.007	-0.008	-0.215	-0.215	-0.062	-0.052	-0.163	-0.085	-0.126	-0.122
Knowledge score (0-5)	1.054§	1.052§	1.119§	1.055‡	1.531§	1.386§	0.913‡	0.910§	0.919†	0.957
	-0.002	-0.002	-0.032	-0.021	-0.018	-0.013	-0.028	-0.022	-0.034	-0.029
Young (15-24 years) (Ref: age >25)	1.009	1.004	1.494§	1.240§	0.909†	0.974	1.186	0.963	1.313†	0.777†
	-0.007	-0.006	-0.115	-0.065	-0.038	-0.032	-0.146	-0.088	-0.182	-0.079
Secondary education or higher	1.099§	1.146§	1.198†	1.539§	2.027§	2.458§	1.043	1.026	0.922	0.935
	-0.012	-0.008	-0.109	-0.078	-0.141	-0.09	-0.162	-0.09	-0.166	-0.087
Working	0.993	0.994	1.041	0.933	1.003	1.031	0.881	0.845†	0.935	0.942
	-0.007	-0.006	-0.081	-0.044	-0.043	-0.031	-0.11	-0.067	-0.136	-0.086
Pregnancy in last 5 years	1.187§	1.190§	1.537§	1.255§	3.468§	3.127§	1.045	1.012	2.079§	2.020§
	-0.008	-0.007	-0.129	-0.059	-0.169	-0.099	-0.117	-0.072	-0.243	-0.174
Wife/co-wife (Ref: head of household)	0.996	0.993	0.802†	0.832‡	0.911	0.914†	1.151	0.956	1.38	1.469‡
	-0.009	-0.008	-0.081	-0.054	-0.053	-0.04	-0.173	-0.096	-0.24	-0.178
Other relationship to head of household	1.028†	1.027†	0.887	1.508§	1.211†	1.026	1.019	0.946	0.856	0.783
	-0.013	-0.011	-0.114	-0.12	-0.091	-0.057	-0.204	-0.127	-0.169	-0.111
Rejects wife-beating	1.044§	1.062§	1.319§	1.233§	1.254§	1.342§	1.124	1.182†	1.121	1.074
	-0.007	-0.006	-0.096	-0.062	-0.048	-0.04	-0.125	-0.093	-0.145	-0.103
Rural (Ref: urban)	0.934§	0.879§	0.731†	0.723§	0.686§	0.406§	1.12	1.075	0.943	1.379§
	-0.015	-0.005	-0.11	-0.034	-0.06	-0.012	-0.257	-0.078	-0.185	-0.123
Constant	12.899§	11.996§	0.024§	0.020§	0.683†	1.165	78.193§	257.842§	188.353§	135.681§
	-0.306	-0.198	-0.007	-0.003	-0.104	-0.143	-30.735	-72.971	-88.364	-45.192
[Pseudo] R-squared	0.428	0.323	0.189	0.169	0.447	0.355	0.100	0.118	0.128	0.153
Number of observations	39** **905	67** **782	36** **342	63** **158	39** **726	67** **461	38** **928	65** **151	39** **665	67** **426

*Notes: Country-fixed effect is not shown in this view. Numbers are odds ratio (standard error). Regressions have been adjusted for HIV status, age group, education level, working status, residence in urban/rural setting, history of pregnancy in past 5 years, relationship to the head of household, rejection of wife beating, and fixed effect for countries. Source: [14]

†*P* < 0.05.

‡*P* < 0.01.

§*P* < 0.001.

The results show that younger women are different in certain aspects of risk: they are more likely to use condoms, but if living in poverty, they are less likely to have ever been tested for HIV compared to older women. Of interest, young age among poor women is associated with single partners, but among richer women, young age becomes a risk factor and is associated with having more than one partner. Across either wealth groups, higher education is associated with higher use of condoms, higher chance of being tested, and better overall behavior score.

Better knowledge is associated with a better overall sexual behavior score, higher condom use, and testing for HIV. With every unit increase in knowledge score, the odds ratio for condom use improves 12% for poor and 6% among non-poor women, and the odds ratio of having been tested for HIV increases 53% for poor vs 39% for non-poor counterparts. However, higher knowledge is associated with 9% increased odds of having STD in both groups and 8% higher odds of having more than one partner among poor women.

Positive associations were also detected for presence of agency with condom use, HIV testing and overall behavior score. Such associations are stronger among those who are not poor and are most prominent in condom use: poor women with agency have 70% higher odds of using condoms compared to poor women who do not have agency. Among women who are not poor, the presence of agency increases the odds of condom use by 130% over the group without agency. With agency, the odds of being tested for HIV increases by 44% among poor and 49% among women who are not poor.

We wanted to understand how a woman’s background of poverty and agency could moderate the effect of knowledge on risk behaviors. **Table 4** shows side-by-side comparisons of separate regression model results that predict behavior as a dependent variable among four groups of women based on wealth and agency. These models were repeated for overall behavior score, condom use, HIV test history, absence of STD, and having a single partner. Absence of agency nullifies the effect of knowledge on condom use regardless of wealth. On the other hand, in presence of agency, knowledge improves the behavior score among women and this effect is stronger among poor women. With each unit increase in knowledge score for poor women who have agency, the odds of condom use increases to 2.11, which is higher in comparison to the odds observed for women who are not poor but have agency at about 1.88. Similarly, knowledge shows a strong association with an absence of STD in these models: 10% higher odds among the non-poor group with agency compared to their counterparts (without agency). The odds for this is only trivially better for poor women when agency exists compared to the poor without agency. Better knowledge enhances the chance of having been tested for HIV across all four groups, but the beneficial effect is greater for those who lack agency (odds of 4.91 vs 4.53 among poor without and with agency, and 4.09 and 3.97 for women who are not poor without and with agency); a 40% difference in odds in the poor group and 10% in the not-poor group. Finally, the results show a negative association of knowledge with having a single partner among women who have agency; agency increases odds of having more than one partner by 10% among both poor and non-poor groups. When all of these behaviors are considered together in the form of a single behavior score, knowledge is associated with better scores across all four groups but shows a smaller association when women have no agency: for poor women with agency, we observed an 8% improvement in behavior score, which decreased to 4% in the group without agency. These results suggest that poor women who do not have agency might not be able to benefit from knowledge as much as their counterparts do.

**Table 4:** Impact of HIV-related knowledge on risk behaviors across 4 groups of married women

Odds ratio	Poor + No agency	Poor + Some agency	Not poor + No agency	Not poor + Some agency
**Behavior score (0-4):**				
Good knowledge (regression coeff.)	1.043§	1.077§	1.046§	1.054§
Standard Error	1.002	1.002	1.003	1.002
**Condom use:**				
Good knowledge (OR)	2.889	3.105§	2.824	2.878‡
Standard Error	1.075	1.036	1.059	1.259
**Been tested**				
Good knowledge (OR)	4.914§	4.531§	4.092§	3.971§
Standard Error	1.036	1.021	1.024	1.016
**No STD:**				
Good knowledge (OR)	2.474†	2.519†	2.401‡	2.514‡
Standard Error	1.042	1.038	1.037	1.31
**Single partner:**				
Good knowledge (OR)	0.924	0.911†	1.05	0.932†
Standard error	1.094	1.042	1.293	1.034

OR – odds ratio, STD – sexually-transmitted disease

*Notes: First row represents regression coefficient for change in behavior score with every unit increase in knowledge score. The following three rows show odds ratio (OR) of adopting safe sexual behavior and outcome (sexually transmitted disease STD) when comprehensive knowledge of HIV is present (knowledge score = 7), ceteris paribus.

Regression coefficients are presented here adjusting for HIV status, age group, education level, working status, residence in urban/rural setting, history of pregnancy in past 5 years, relationship to the head of household, and fixed effect for countries. Coeff. Denotes coefficient. Source: [14].

†*P* < 0.05.

‡*P* < 0.01.

§*P* < 0.001.

## DISCUSSION

Knowledge of HIV is a clear protective factor for married women, but lack of agency diminishes its effect, and when coupled with poverty, there is evidence that knowledge provides no further protection. Looking at direct effects, in both groups of women living in poverty and those who are not poor, knowledge showed positive associations with a better score in sexual behavior, specifically with a higher rate of condom use and HIV testing. Similarly, our results show a direct effect for women’s personal agency on better behavior score, condom use and opting for HIV testing. We investigated the relationship between knowledge and behavior under a background of different combinations of poverty and agency. We observed a mix of effects that shows complex interactions between availability of knowledge, presence of agency, and poverty. In summary, these analyses suggest that better knowledge may not be as effective as might be expected when agency is lacking. When poor women have no agency, knowledge does not show any effect on condom use. On the other hand, if women do have some level of agency, knowledge seems to have an overall stronger impact among poor women (condom use, no history of STD and improved behavior score). Unlike the effect on condom use and STD, better knowledge showed a stronger effect on increased adoption of HIV testing among women who lack agency, although improvement in behavior is seen in all four groups. Finally, we observed a negative association between better knowledge and having single partners when women have some agency. However, the positive effect of knowledge and agency on testing, condom use, and absence of STD are much larger than the observed negative association with having a single partner. The combination of all these effects moves the overall behavior score in the desired positive direction and is indicative of potential success for educational campaigns in promotion of safe sex behavior.

The group of women with poverty and lacking agency was disproportionately less educated (only 3.3% had secondary education or higher) and a staggering 17% had not heard of HIV/AIDS. Only half of poor women with no agency have comprehensive knowledge about HIV prevention or mother-to-child transmission, compared to an overall average above 60% among women with agency. This points to lack of access to information. Since this group mainly resides in rural areas, one has to consider the efficacy of outreach and coverage in areas where cultural and socioeconomic circumstances create barriers for successful interventions. Other studies also found positive associations between higher education, higher household wealth, living in urban areas, and women's better knowledge [16,17]. The relationship between economic status, women’s empowerment, and behavior has been noted before. As an example, economic empowerment of young women through cash transfer in Tanzania has shown to enhance self-esteem and confidence in decision-making and reduced transactional sex, intimate partner violence, and overall risky behaviors [18]. Besides a lower degree of protective factors, we demonstrated that a combination of poverty and restricted agency moderates the effect of knowledge on behaviors in a negative way. This puts poor women with no agency in a particularly vulnerable position.

While seemingly counterintuitive, poorer women and those without agency had lower rates of HIV seropositivity, STDs, and having multiple partners. These findings are not surprising, as other studies have shown similar trends. For instance, in sub-Saharan African countries, HIV prevalence was positively associated with indicators of women's empowerment such as enrollment in secondary education [6]. Similarly, higher rates of partner change was reported to link with education and wealth [19]. Women who are empowered with wealth and education in Gabon, Mozambique, Sierra-Leone and Zambia are shown to have a higher likelihood to engage in riskier behaviors [20]. It is more likely that the correlation between wealth and risky behavior is a nonlinear form, or J-shaped, with those in the two ends of the spectrum being particularly at risk [21]. Regardless, one should not assume that the lower rate of HIV and STD among married poor women with no agency makes them less of a vulnerable group because an overly simplified view of risk has shown its unfortunate consequences. As a case in point, while historically married women were considered low risk for HIV and overlooked in many nation-wide programs [22], recent reports show that marriage is no longer necessarily a protective factor [23]. In Ethiopia, a 2011 survey showed that married women, probably infected through their spouses, are infected at a rate three times the rate for women who have never been married [24]. This is an opposite pattern from what was shown in a 2005 survey, and scholars have attributed this trend to the fact that married women have not benefited from anti-HIV interventions as much as their unmarried, younger counterparts [24].

With all the complexities that have been described at the intersection of poverty, lack of agency, and anti-HIV campaigns, programs with a more systematic approach in addressing the social, financial, and educational barriers that their target population face will have a better chance to succeed. Incorporating social programs with special attention to women’s empowerment for both economic control and building self-advocacy skills should supplement health promotion programs. In many situations, this becomes a development program rather than merely a health-sector initiative. As an example of such multi-faceted initiatives, there is a report from a proposed structural intervention in Botswana that tackles a range of social and economic factors with the goal of reducing the vulnerability of young women that eventually fights HIV transmission. These intermittent interventions cover issues such as gender inequality, gender violence, poverty, and poor access to education. The ability of women to make choices and assert their position in their household and in the community are among the objectives of this program [25]. The Sonagachi Project in India is an example of a successful program that directly targeted self-efficacy and agency for sex workers. This program aimed to specifically build community empowerment through collective identity, raising awareness and providing negotiation skills, as well as educating women on HIV-related issues [25] and it eventually succeeded in promoting condom use and safer sexual practices.

While a more detailed analysis with robust quantitative methods is definitely warranted, we should note that it is difficult to ‘*quantify*’ when and how education and higher economic well-being can lead to the adoption of safer practices such as condom use to an extent that it could offset risk of some women taking multiple partners. Therefore, theories backed by evidence from quantitative studies should be supplemented by qualitative field studies in order to inform local and national policy. Such research should examine drivers of behavior within specific sociocultural backgrounds to understand which types of barriers exist in communities that weaken the effectiveness of educational campaigns. It is also important to extend similar research to medication adherence such as ART, where treatment success is contingent upon continuous compliance to a prescribed drug.

### Policy implications

Success of HIV knowledge-building campaigns cannot be divorced from the contextual factors of individual women’s lives. We demonstrated that an environment afflicted by poverty and a lack of self-efficacy is perilous to the successful implementation of anti-HIV campaigns. We demonstrated that women who are living in poverty and lack agency show a distinct profile that affects their risk, including lower awareness of HIV, or knowledge of HIV prevention and transmission. Furthermore, without agency, the impact of knowledge is attenuated in the successful adoption of several safe sexual practices, but when agency exists, knowledge can be more influential among poor women compared to non-poor counterparts. This is an invitation to interventions that systematically address the sociocultural and structural factors that put women in a powerless position, further plagued by financial insecurity.

### Limitations of the research

The major limitation of this study is the cross-sectional nature of the study that prevents us from drawing causal conclusions. Given the complex nature of ways in which individuals make decisions about their behaviors, and dynamics between behavior-outcome-change of behavior, it is difficult to see the cause-and-effect relationships.

With regards to missing values for the questions on ‘agency’, we had to make a decision on imputation of the missing values vs exclusion of non-responders. Since potentially there are unobservable cultural and social factors causing the respondent to avoid particular questions (especially considering the diversity of the subjects in the study), we decided against the imputation and omitted observations that had missing response on agency. This will be a source of bias in calculation of rates for levels of agency which was not a focus of current study. Nonetheless, we advise for caution in generalizing the findings of association analyses. Further studies with more scrupulous sampling design are warranted.

Lastly, another limitation of the study (and opportunity for future research) is to repeat similar analyses among unmarried women. Unfortunately, our sample did not allow us to examine unmarried women because the DHS survey questions were not posed to that group.

## Additional material


Online Supplementary Document
