# Supplementary material for: Between a Rock and a Hard Place: How poverty and lack of agency affect HIV risk behaviors among married women in 25 African countries: A cross-sectional study
Source: J Glob Health. 2021 Oct 30;11:04059. doi: 10.7189/jogh.11.04059 (PMC8564885; doi:10.7189/jogh.11.04059)
Supplement: Online Supplementary Document [file jogh-11-04059-s001.pdf]

Online Supplementary Document- Tables S1 and S2

Table S1. Details of questions used in construction of study variables.

Variable and the composites	Description of DHS questions
**Awareness of HIV/AIDS**	Whether the respondent has ever heard of AIDS (Acquired Immune Deficiency Syndrome)
**Knowledge of HIV prevention methods**	Correct answer to both questions:<br>Respondents were asked whether not using condoms would reduce their chances of getting AIDS<br>Respondents were asked whether having just one sexual partner would reduce their chances of getting AIDS
**Comprehensive knowledge of mother-to-child transmission**	Correct answer to all 3 questions on ways of transmission from mother to child:<br>During pregnancy<br>During delivery<br>By breastfeeding
**Sexual agency**	Positive response to at least one of these questions:<br>respondent can refuse sex<br>respondent can ask for condom use if husband has STD
**Agency in household decision making**	A response indicating 'myself' or 'myself and my husband' for at least one of these questions:<br>Person who should have greater say on how to spend money<br>Person who should have greater say on making large household purchases<br>Person who should have greater say on responder's own health
**Refusal of wife beating**	A response indicating 'wife beating not justified' to ALL of these questions:<br>Beating justified: wife neglects children<br>Beating justified: wife goes out without telling husband<br>Beating justified: wife refuses to have sex with him
**Behavior-1 (use of protection)**	Used condom in last sexual intercourse
**Behavior-2 (previous testing)**	Ever been tested for AIDS
**Behavior-3 (absence of history of STD)**	Did not report any Sexually Transmitted Diseases over the last 12 months, or abnormal genital discharge, or genital ulcer
**Behavior-4 (single partners)**	If Number of sexual partners, including husband during the last 12 months <=1
**Agency**	A positive value for both sexual agency and agency in household decision making
**KNOWLEDGE SCORE**	1 score for each correct answer to these questions:<br>Using condoms would reduce their chances of getting AIDS<br>Having just one sexual partner would reduce their chances of getting AIDS Pregnancy is a way of HIV transmission from mother to child<br>Child delivery is a way of HIV transmission from mother to child<br>Breastfeeding is a way of HIV transmission from mother to child
**BEHAVIOR SCORE**	1 score for each positive answer to these items:<br>Used condom in last sexual intercourse<br>Ever been tested for AIDS<br>absence of history of STD during the last 12 months<br>having single partner during the last 12 months

Notes: DHS denotes Demographic and Health Surveys; STD denotes sexually transmitted disease; HIV/AIDS denotes human immunodeficiency virus infection / acquired immune deficiency syndrome.

Table S2. Awareness of HIV/AIDS among adult married women across different countries by level of poverty.

	Poor + No Agency		Poor + Some Agency		Not Poor + No Agency		Not Poor + Some Agency		Total		Number of observations
Country	%	SE	%	SE	%	SE	%	SE	%	SE	n
Angola	36.4%	4.2%	54.5%	2.2%	74.1%	4.7%	89.7%	1.1%	77.2%	1.2%	7,172
Burkina Faso	97.7%	0.4%	98.8%	0.5%	99.4%	0.2%	99.7%	0.1%	99.1%	0.2%	6,656
Burundi	93.5%	1.6%	96.8%	0.5%	97.0%	0.9%	98.4%	0.3%	97.4%	0.3%	4,822
Cameroon	82.4%	2.5%	93.7%	1.6%	96.9%	1.1%	99.5%	0.2%	95.5%	0.6%	4,529
Chad	72.0%	2.1%	89.6%	1.3%	71.8%	1.7%	89.9%	1.1%	80.5%	1.0%	4,391
Congo Democratic Rep	81.8%	2.4%	91.2%	1.1%	90.1%	2.2%	96.9%	0.5%	93.2%	0.7%	5,946
Cote d'Ivoire	84.9%	3.3%	93.6%	1.6%	90.5%	2.4%	97.5%	0.9%	93.4%	1.0%	2,876
Ethiopia	85.8%	1.8%	87.2%	2.0%	94.2%	1.2%	95.9%	0.6%	92.8%	0.7%	9,789
Gabon	89.2%	3.6%	96.2%	0.8%	95.7%	2.1%	99.6%	0.3%	98.8%	0.3%	2,866
Gambia	92.4%	2.5%	98.4%	0.5%	96.8%	1.3%	99.3%	0.2%	98.2%	0.4%	2,951
Ghana	85.3%	3.9%	94.3%	1.4%	98.3%	1.4%	99.2%	0.2%	97.6%	0.4%	2,652
Guinea	88.7%	2.0%	91.4%	1.6%	95.9%	0.8%	97.4%	0.6%	94.3%	0.7%	3,408
Lesotho	88.5%	5.6%	98.1%	0.5%	97.0%	3.0%	99.8%	0.1%	99.1%	0.2%	1,775
Liberia	78.2%	6.4%	93.5%	1.0%	88.7%	3.7%	98.5%	0.3%	96.3%	0.4%	2,505
Malawi	95.5%	1.3%	98.0%	0.5%	91.6%	2.4%	99.3%	0.2%	98.1%	0.3%	5,220
Mali	68.9%	2.4%	85.0%	2.6%	82.1%	1.5%	90.8%	1.5%	80.1%	1.3%	4,333
Mozambique	88.5%	2.6%	93.6%	1.0%	91.7%	1.8%	97.7%	0.5%	95.0%	0.7%	4,561
Namibia	93.5%	4.8%	98.7%	0.5%	91.6%	8.8%	99.9%	0.1%	99.4%	0.2%	1,360
Niger	77.5%	2.1%	83.2%	2.4%	84.8%	1.6%	94.1%	1.0%	84.5%	1.2%	4,527
Rwanda	100.0%	0.0%	100.0%	0.0%	100.0%	0.0%	100.0%	0.0%	100.0%	0.0%	3,522
Senegal	86.5%	1.9%	92.7%	1.3%	96.7%	0.8%	98.0%	0.7%	94.9%	0.6%	3,712
Sierra Leone	84.9%	2.2%	92.7%	1.3%	90.6%	1.5%	95.9%	0.7%	92.8%	0.9%	5,215

	Poor + No Agency		Poor + Some Agency		Not Poor + No Agency		Not Poor + Some Agency		Total		Number of observations
Country	%	SE	%	SE	%	SE	%	SE	%	SE	n
**Togo**	88.4%	2.6%	96.3%	0.8%	96.1%	1.4%	99.0%	0.2%	97.4%	0.4%	3,163
**Zambia**	97.7%	0.8%	99.4%	0.2%	99.9%	0.1%	99.9%	0.1%	99.6%	0.1%	9,249
**Zimbabwe**	97.0%	1.5%	99.2%	0.2%	100.0%	0.0%	99.8%	0.1%	99.5%	0.1%	5,550
**Total**	83.0%	0.6%	92.3%	0.3%	90.0%	0.4%	97.4%	0.1%	93.6%	0.2%	**112,749**

Notes: SE denotes standard error; HIV/AIDS denotes human immunodeficiency virus infection / acquired immune deficiency syndrome.

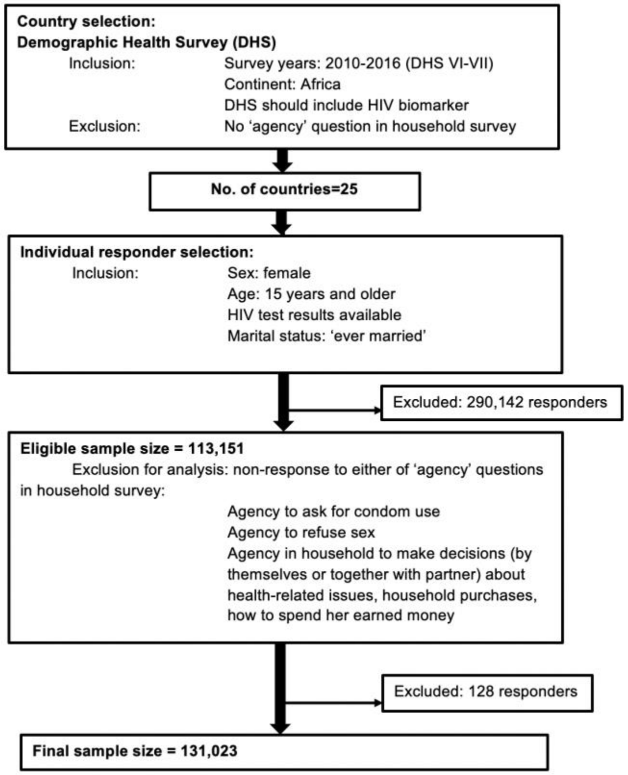

Figure 1: Flow-chart depicting shows sample selection process in this study.

Commented [CH1]: Are the numbers in the correct in this figure? How did you go from 113,151, then exclude 128 and come up with 131,023? Is it OK that you haven't shown that at the starting point the number of responders? Or is that not so important?

Corresponding Author: Roya Sherafat-Kazemzadeh, MD, PhD, Institute for Global Health and Development, The Heller School for Social Policy and Management, Brandeis University, 415 South Street, MS035, Waltham Massachusetts 02453, USA. Tel: 781-736-3919; email: sherafat@brandeis.edu.
